# Supplementary material for: In Silico Whole Genome Sequencer and Analyzer (iWGS): a Computational Pipeline to Guide the Design and Analysis of de novo Genome Sequencing Studies
Source: G3 (Bethesda). 2016 Sep 15;6(11):3655–62. doi: 10.1534/g3.116.034249 (PMC5100864; doi:10.1534/g3.116.034249)
Supplement: Supplemental Material [file supp_6_11_3655__index.html]

In Silico Whole Genome Sequencer and Analyzer (iWGS): a Computational Pipeline to Guide the Design and Analysis of de novo Genome Sequencing Studies — Supplemental Material 

# *In Silico* Whole Genome Sequencer and Analyzer (iWGS): a Computational Pipeline to Guide the Design and Analysis of *de novo* Genome Sequencing Studies

## Supplemental Material for Zhou, *et al*, 2016

**Files in this Data Supplement:**

- Table S1 - Characteristics of genomes selected for case studies. (.xlsx, 13 KB)
- Table S2 - Complete QUAST report for *de novo* assemblies generated in the Case study I (best-performing strategy highlighted in light green). (.xlsx, 47 KB)
